# High Dose Intravenous IgG Therapy Modulates Multiple NK Cell and T Cell Functions in Patients With Immune Dysregulation

**DOI:** 10.3389/fimmu.2021.660506

**Published:** 2021-05-19

**Authors:** Sarah M. McAlpine, Sarah E. Roberts, John J. Heath, Fabian Käsermann, Andrew C. Issekutz, Thomas B. Issekutz, Beata Derfalvi

**Affiliations:** ^1^ Department of Pediatrics, Dalhousie University, Halifax, NS, Canada; ^2^ CSL Behring Research, CSL Biologics Research Center, Bern, Switzerland

**Keywords:** IVIG, NK cell, immune dysregulation, Kawasaki disease, autoimmune disease

## Abstract

Intravenous immunoglobulin (IVIG) is an effective immunomodulatory treatment for immune dysregulation diseases. However, the mechanisms by which it reduces systemic inflammation are not well understood. NK cell cytotoxicity is decreased by IVIG in women with reduced fertility, but IVIG effects on NK cells in immune dysregulation are less clear. We hypothesized that IVIG modulation of lymphocyte function, especially in NK cells, is important for resolution of inflammation. Our aim was to identify IVIG-induced changes in a cohort of patients with Kawasaki disease (KD) and those that occur broadly in pediatric patients with various immune dysregulatory diseases. Peripheral blood mononuclear cells (PBMCs) of patients with KD or autoimmune/inflammatory diseases were phenotyped pre and post high dose IVIG treatment by flow cytometry. In KD patients, after IVIG infusion T_reg_ cell frequency and the proportion of activated CD25^+^ immunoregulatory CD56^bright^ NK cells was increased, and multiple lymphocyte subsets showed increased expression of the lymphoid tissue homing receptor CD62L. Importantly, IVIG treatment decreased the frequency of cells expressing the degranulation marker CD107a among cytotoxic CD56^dim^ NK cells, which was reflected in a significant reduction in target cell killing and in decreased production of multiple pro-inflammatory mediators. Interestingly, the activating receptor CD336 was expressed on a higher proportion of CD56^bright^ NK cells after IVIG in both KD and autoimmune/inflammatory patients while other NK receptors were increased differentially in each cohort. In autoimmune/inflammatory patients IVIG induced the proliferation marker CD71 on a higher percentage of CD56^dim^ NK cells, and in contrast to KD patients, CD107a^+^ cells were increased in this subset. Furthermore, when PBMCs were stimulated *ex vivo* with IL-2 or *Candida* antigen in autologous plasma, more of the CD4^+^ T cells of KD patients expressed CD25 after IVIG therapy but fewer cytotoxic T cells were degranulated based on CD107a expression. In summary, IVIG treatment in patients with immune dysregulation has multiple effects, especially on NK cell subsets and CD4^+^ T cells, which are compatible with promoting resolution of inflammation. These novel findings provide insight into the immunomodulatory actions of IVIG in autoimmune and inflammatory conditions.

## Introduction

Intravenous immunoglobulin (IVIG) is used at low doses as replacement therapy in patients with primary immunodeficiencies, while at high doses it is a potent immunomodulator to reduce morbidity in hyperinflammatory conditions such as Kawasaki disease (KD) and in autoimmune diseases such as immune thrombocytopenia [reviewed in (1)]. IVIG is also used in women with recurrent miscarriage or implantation failure to enable full-term pregnancy (2, 3). In patients with KD, high dose (2 g/kg) IVIG infusion results in remarkable reduction of fever and laboratory indicators of inflammation such as C-reactive protein (4). However, the mechanism(s) by which IVIG induces its immunomodulatory effects are not completely understood. Many studies have attempted to elucidate the effects of IVIG on inflammatory processes, and possible mechanisms include saturation/modulation of Fcγ receptors, inhibition of cytokines, modulation of endothelium phenotype, neutralization of superantigen and/or bacterial components, anti-idiotype inhibition of auto-antibodies, modulation of B cell and natural killer (NK) cell activity, inhibition of the complement cascade, and enhancement of regulatory T (T_reg_) cell function [reviewed in (5, 6)].

It is likely that a major action of IVIG is by binding to Fc receptors on immune cells, such as B cells, monocytes/macrophages, NK cells, neutrophils and mast cells, and this binding alters the function of such cells. NK cells express Fcγ receptors and can induce cytotoxicity in target cells *via* signals transmitted through Fcγ receptors (antibody-dependent cell-mediated cytotoxicity, ADCC) or *via* a variety of activating and inhibitory receptors (natural cytotoxicity) expressed on the cell surface. CD56^bright^CD16^−^ NK cells compose ~10% of peripheral NK cells and are thought to have an immunoregulatory role based on their ability to secrete cytokines, migrate to lymph nodes and tissues, and expand in pregnancy where they mediate immune tolerance [reviewed in (7)]. CD56^bright^ NK cells can promote or suppress T cell activity [reviewed in (8, 9)], which may be achieved through receptor-ligand interactions (such as CD94/NKG2D) or secretion of soluble mediators (such as interferon (IFN)-γ or interleukin (IL)-10). In contrast, the major peripheral subset CD56^dim^CD16^+^ NK cells are well granulated and are cytotoxic, readily recognizing and killing infected and transformed cells.

IVIG modulation of NK cells has been demonstrated in both *in vivo* and *in vitro* studies showing that IVIG can affect NK cell frequency and phenotype in the blood, and can also alter NK cell cytokine production, proliferation and cytotoxicity (10–15). However, there is a lack of consensus on whether IVIG activates or inhibits NK cells. Additionally, few studies have examined the mechanism by which IVIG alters NK cell function, with almost none focusing on patients with KD. Finberg et al. showed that NK cells from KD patients had enhanced natural cytotoxicity and ADCC after IVIG infusion (16). Furthermore, the activating receptor NKG2D is decreased on NK cells and CD8^+^ T cells in KD patients, and IVIG can restore NKG2D expression in a subset of patients (17). In women with recurrent miscarriage who were treated with IVIG, NK cell cytotoxicity was diminished after treatment, which coincided with increased inhibitory and decreased activating NK receptor expression (10). These studies clearly demonstrate that NK cells respond to IVIG, but the mechanism by which IVIG attenuates hyperinflammation across multiple disorders is not understood, and the role of NK cells in that process has not been fully investigated.

This study explored the quantitative, phenotypic and functional changes in NK cell and T cell subsets that occurred 24-48 hours after infusion with an immunomodulatory dose of IVIG in patients with immune dysregulation. We hypothesized that IVIG induces inhibition of lymphocyte activation, proliferation and cell trafficking, especially in NK cells, which is important for resolution of inflammation in patients with immune dysregulation. Specifically, we were interested in determining whether certain IVIG effects are specific to a well-defined KD patient cohort, or occur globally in all patients studied. Our findings indicate that after IVIG treatment of patients the proliferation, activation and expression of effector molecules by specific NK and T cell subsets are modulated and differentially affected in patients with KD and those with various autoimmune/inflammatory conditions. Furthermore, modulation of these cell types was also demonstrable during *in vitro* stimulation and culture of PBMCs in autologous plasma from KD patients collected post-IVIG therapy.

## Materials and Methods

### Study Participants

The study was approved by the Research Ethics board of the IWK Health Centre, Nova Scotia, Canada, and informed consent was obtained from all patients or their parents/guardians. Twenty-three patients (10 females, 13 males, range 2-16 years old, mean 8.2 ± 4.3 years old) diagnosed with KD or with autoimmune/inflammatory diseases received a high, immunomodulatory dose (1-2 g/kg/dose) of IVIG. The IVIG products administered were Privigen (CSL Behring) to 13 patients, Octagam (Octapharma) to eight patients, Gamunex (Grifols) to one patient, and Gammagard (Shire Pharma) to one patient, as made available by Canadian Blood Services for such therapy. Eleven of the 23 patients had typical KD (18) with no comorbidities and were on no chronic medications except one patient who took Singulair for asthma (**Table 1**). KD patients received only acetaminophen or ibuprofen for fever prior to IVIG therapy. All but one KD patient received a single dose of IVIG (2 g/kg), all were treated with 100 mg/kg/day ASA starting at the time of IVIG infusion which was decreased to 3-5 mg/kg/day when the patient became afebrile, and all patients fully recovered. One patient had mild coronary artery ectasia. One patient developed a right coronary artery aneurysm, which resolved after a second dose of IVIG (2 g/kg).

**Table 1:** Kawasaki disease patient characteristics.

Parameter	KD
No. of cases	11
Age, years (mean ± SD)	5.4 ± 1.9
Sex (M/F)	5/6
C-reactive protein [mg/L]	
Pre-IVIG	89.4 ± 66.9
Post-IVIG	32.1 ± 30.0**
Absolute lymphocyte count [10^9^/L]	
Pre-IVIG	2.4 ± 1.1
Post-IVIG	2.6 ± 1.3
Typical KD^1^	11/11 (100%)
Coronary artery abnormalities	2/11 (18%)

^1^Patients had fever for five or more days with at least four of five principal clinical features: bilateral conjunctival injection, changes in the lips and oral cavity, cervical lymphadenopathy, extremity changes, and polymorphous rash. **p < 0.01.

**Table 2** shows the characteristics of all other patients who had various autoimmune/inflammatory diseases and were administered 1-2 g/kg/dose IVIG. Two patients received immunosuppressive medications at the time of and for at least 5 days before blood collection. Ten of the 12 patients showed a clinical response to IVIG infusion within days followed by gradual improvement of their autoimmune/inflammatory condition.

**Table 2:** Characteristics of patients with autoimmune/inflammatory diseases.

Patient	Age (years)	Sex	Condition	Medications	Comorbidities
1	12	M	CNS inflammation (PANS)	–	Food allergies, hay fever, allergic asthma
2	16	F	Autoimmune neuropathy/channelopathy	Naprosyn, Ranitidine, Keppra	Raynaud, arthralgia
3	11	M	AIHA	Prednisolone	–
4	5	M	CNS inflammation (CNS small vessel vasculitis)	Ranitidine	–
5	8	M	CNS inflammation (ADEM)	–	–
6	12	M	CNS inflammation (PANS)	–	Mild hypogammaglobulinemia
7	15	F	Guillain-Barré syndrome	–	–
8	11	M	Dermatomyositis	Methotrexate, ranitidine	–
9	7	F	ITP	Clonidine, domperidone, lansoprazole, ferrous sulfate, trimethoprim/sulfamethoxazole, nitrofurantoin	Anoxic brain injury, global developmental delay, neurogenic bladder, urosepsis
10	15	M	CNS inflammation (chorea)	Amytriptiline	Juvenile idiopathic arthritis
11	15	F	SLE	Medroxyprogesterone acetate	–
12	2	M	Systemic JIA	–	–
Mean ± SD	10.8 ± 4.3	8/4 (M/F)			

CNS, central nervous system; PANS, Pediatric Acute onset Neuropsychiatric Syndrome; AIHA, autoimmune hemolytic anemia; ADEM, Acute disseminated encephalomyelitis; ITP, immune thrombocytopenic purpura; SLE, systemic lupus erythematosus; JIA, juvenile idiopathic arthritis.

### Blood Sample Processing and Cryopreservation

Peripheral blood samples were collected before and 24-48 hours after IVIG infusion. Heparinized blood was centrifuged for 20 min at 400g to obtain plasma, which was then centrifuged for 20 min at 2400g to eliminate platelets before freezing at -80°C. The cell pellet was resuspended in HBSS (Gibco, Gaithersburg, MD) containing 15 mM HEPES (Gibco) and 0.1% human serum albumin (HSA), layered over Ficoll-Paque PLUS (GE Healthcare, Uppsala, Sweden), and centrifuged at 400g for 30 min. Peripheral blood mononuclear cells (PBMCs) were collected, washed and cryopreserved in RPMI-1640 (Gibco) containing 50% human AB plasma and 10% dimethyl sulfoxide (Sigma-Aldrich, St. Louis, MO) and stored in liquid nitrogen. Dimethyl sulfoxide was added over a five minute period, which allows recovery of NK cell cytotoxicity comparable to that of freshly isolated NK cells (19) as observed also by us (unpublished observations). These optimal methods of freezing PBMCs allowed recovery of 70-80% of cells with >90% viability and no significant change in proportion of lymphocyte subsets.

### Culturing Conditions

PBMCs were thawed, washed with HBSS containing 15 mM HEPES and 0.1% HSA, centrifuged at 300g for 10 min, resuspended and cell viability was determined by trypan blue exclusion. Autologous plasma was thawed on ice and centrifuged at 1500g for 10 min at 4°C to remove cryoprecipitates. Cells intended for *ex vivo* characterization *via* flow cytometry were removed and stained immediately as described below. The remaining cells were stimulated in 24-well plates with a sub-mitogenic concentration of IL-2 (30 IU/mL; Peprotech, Rocky Hill, NJ) for 3 days or *Candida albicans* extract (50 PNU/mL; Omega Laboratories, Montreal, QC, Canada) for 6 days. The cells were stimulated at a concentration of 2x10^6^ cells/mL in RPMI-1640 supplemented with 50% autologous plasma collected at the same time point. This plasma concentration was chosen to try to recapitulate during culture the milieu of the mononuclear cell *in vivo* i.e. the presence of all plasma factors and mediators, and the infused IgG in the case of samples taken post IVIG treatment. After stimulation, the cells were stained with antibodies as described below.

### NK Cell Cytotoxicity Assay by Flow Cytometry

To prepare effector cells, PBMCs isolated pre- and post-IVIG infusion were incubated overnight in RPMI plus 25% autologous plasma obtained at the same time point, which reverses any artificial inhibition of NK cell cytotoxicity caused by cryopreservation (19). To eliminate the possibility of ADCC resulting from IVIG binding to K562 target cells (14), effector cells were washed twice with HBSS containing 0.1% HSA after overnight incubation in autologous plasma. The erythroleukemia K562 cell line, which does not express MHC-I (20), was used as a target cell population, and was pre-labeled with 0.35 µg/mL carboxyfluorescein diacetate (Sigma-Aldrich) in PBS for 30 min, washed, and resuspended in RPMI containing 10% fetal bovine serum. PBMCs were combined with K562 cells at an effector:target cell ratio of 20:1 and incubated for 4 hours in RPMI containing 10% fetal bovine serum (Gibco). In some tubes, brefeldin A (eBioscience) was added after one hour to inhibit protein transport to the surface and co-cultures were incubated for five hours total. After the incubation, co-cultures were washed and labelled with antibodies as described below. K562 cells were identified by their carboxyfluorescein diacetate fluorescent emission, and killed cells were determined by their uptake of a fixable viability dye (**Table S1**). For each assay run, a “spontaneous death” control sample was also prepared in order to subtract the % spontaneous K562 death from the % killed in each patient assay. NK specific cytotoxicity was calculated by determining the true effector:target ratio (total NK cells:total K562 cells), and dividing this ratio into the percentage of killed K562 cells, as follows: (% Dead K562 in Co-culture - % Spontaneous K562 Death) ÷ (# of CD56^+^ CD3^−^ events/# K562 events).

### High Dimensional Flow Cytometry

PBMCs that were freshly thawed, stimulated with IL-2 or *Candida* extract, or co-cultured with K562 cells were first washed with immunofluorescence buffer containing Dulbecco’s PBS (Gibco), 0.5% BSA (Sigma-Aldrich) and 0.1% NaN3. Fc receptors were blocked with heat-aggregated human IgG for 15 min on ice. PBMCs were then labelled with antibody panels designed to characterize NK cell and T cell subset immunophenotype and activation state for 15 min at room temperature, then washed once with PBS. **Table S1** shows the antibodies and reagents used for immunofluorescent staining. Dead cells were labelled with a fixable viability dye for 30 min on ice. For PBMCs undergoing surface staining only, cells were washed with immunofluorescence buffer and fixed in 1% paraformaldehyde. For intracellular cytokine staining and FoxP3 labelling, cells were washed with immunofluorescence buffer after surface staining, then fixed, permeabilized and stained with antibody using the FoxP3 Transcription Factor Staining Buffer Set according to manufacturer’s instructions (eBioscience). Immunofluorescently labeled PBMCs were acquired on a BD LSRFortessa (BD Biosciences, St. Jose, CA). All spins were performed at 700xg for 5 min.

### Measurement of Inflammatory Cytokines and Chemokines

The concentration of various cytokines and chemokines in patient plasma and in culture supernatants from IL-2 and *Candida* stimulation of PBMCs was measured on a Bio-Plex 200 instrument (Bio-Rad), using a custom designed Magnetic Luminex Performance Assay according to the manufacturer’s instructions (R&D Systems, Minneapolis, MN). Data analysis was performed using Bio-Plex Manager software v6 (Bio-Rad).

### Data Analysis

Flow cytometry data were analyzed using FlowJo version 10.5.3 for Windows (Becton Dickinson, Ashland, OR). Statistical analysis of pre- and post-IVIG data from each cell subset was performed using GraphPad Prism version 7.05 for Windows (GraphPad Software, San Diego, CA). Individual comparisons were deemed significant where p < 0.05 by paired Student’s t-test, except where data were not normally distributed and were analyzed by Wilcoxon paired rank test.

## Results

### IVIG Induced the Activation and Proliferation of NK Cell and T Cell Subsets

**Figure 1:**
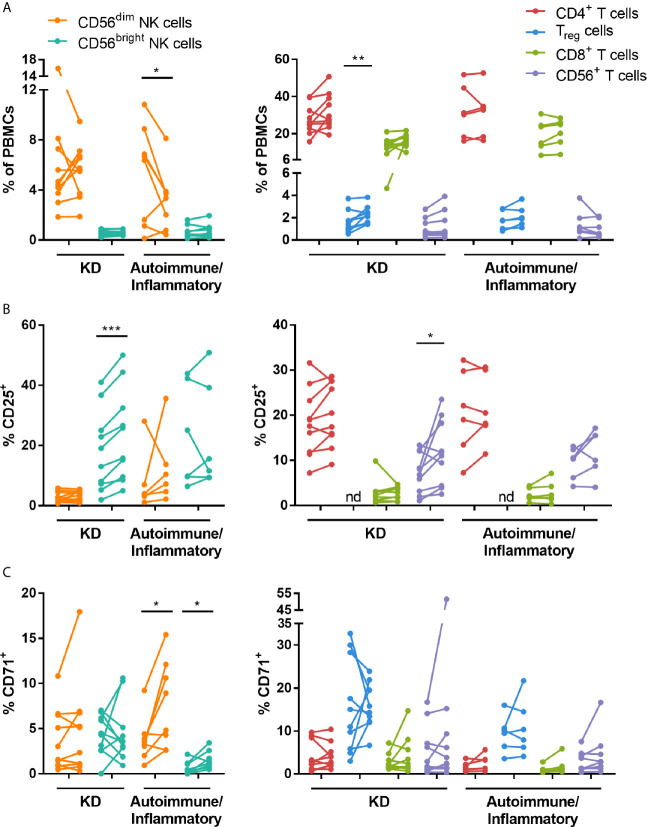
High dose IVIG infusion selectively modulates the frequency, activation and proliferation of NK cell and T cell subsets *in vivo*. PBMCs obtained pre- and post-IVIG infusion from KD patients (n=10) and autoimmune/inflammatory patients (n=6-8) were analyzed by immunofluorescence and multiparameter flow cytometry for **(A)** NK cell and T cell subset frequency among PBMCs, **(B)** the activation marker CD25 and **(C)** the proliferation marker CD71. Graphs show individual paired samples with lines connecting pre-IVIG (left) and post-IVIG (right) data. *p < 0.05; **p < 0.01; ***p < 0.001; nd, not determined.

To investigate the effects of IVIG on the phenotype and function of NK cells and T cells, PBMCs isolated pre- and 24-48 hours post-IVIG infusion from patients with KD or autoimmune/inflammatory diseases were characterized by multiparameter flow cytometry. An example gating strategy used to assess the activation and proliferation of multiple NK cell and T cell subsets is shown in **Figure S1**. In KD patients, the frequency of T_reg_ cells was significantly increased in the post-IVIG blood samples (**Figure 1A**), and IVIG treatment enhanced the activation status of both CD56^bright^ NK cells and CD56^+^ T cells based on the proportion of CD25^+^ cells (**Figure 1B**). In autoimmune/inflammatory patients, CD56^dim^ NK cells were decreased in frequency among PBMCs, but the proliferation marker CD71 was expressed on a higher percentage of both CD56^dim^ and CD56^bright^ NK cells (**Figures 1A, C**).

### IVIG Induced Changes in Plasma Cytokines

Patient plasma cytokine measurements showed that in KD patients, IL-6 (p = 0.02) and the chemokine CXCL10 (p = 0.008) were significantly decreased after IVIG infusion while IL-4 (p = 0.013) was modestly increased (**Table 3**). In contrast, CXCL10 was increased in patients with autoimmune/inflammatory diseases (p = 0.006) after IVIG. Several other pro- and anti-inflammatory mediators were unchanged.

**Table 3:** Plasma cytokine concentrations in KD and autoimmune/inflammatory diseases.

Analyte	Kawasaki Disease (n = 9)		Autoimmune/Inflammatory Diseases (n = 10)	
	Pre-IVIG	Post-IVIG	Pre-IVIG	Post-IVIG
IL-1β	1.6 ± 0.3	1.1 ± 0.2	0.8 ± 0.3	1.0 ± 0.3
IL-4	1.3 ± 0.2	1.8 ± 0.2*	8.7 ± 6.0	2.8 ± 1.3
IL-6	88.4 ± 49.1	16.3 ± 3.5*	29.5 ± 12.0	45.9 ± 17.7
IL-7	3.4 ± 1.1	4.0 ± 1.3	6.1 ± 3.8	3.8 ± 1.8
IL-10	5.6 ± 1.7	2.2 ± 0.4	0.7 ± 0.1	1.1 ± 0.2
IL-12p70	2.0 ± 0.9	3.2 ± 1.1	3.0 ± 0.5	3.1 ± 0.7
IL-13	3.0 ± 0.9	2.1 ± 0.7	2.3 ± 0.4	1.5 ± 0.4
IL-15	3.3 ± 0.7	3.1 ± 1.0	4.5 ± 2.1	3.3 ± 1.1
IL-17F	4.1 ± 1.7	1.1 ± 0.5	1.4 ± 0.7	1.1 ± 0.4
IL-33	1.4 ± 0.5	1.2 ± 0.4	0.5 ± 0.2	0.5 ± 0.2
IFN-α	31.1 ± 7.4	21.5 ± 8.4	36.9 ± 17.4	13.2 ± 6.9
IFN-γ	1.8 ± 1.4	2.8 ± 1.5	0.6 ± 0.6	0.6 ± 0.6
GM-CSF	0.5 ± 0.2	0.3 ± 0.2	0.4 ± 0.1	0.3 ± 0.1
Granzyme B	33.1 ± 7.8	18.9 ± 3.2	5.8 ± 1.7	5.8 ± 1.1
PDGF-AB/BB	1103.4 ± 315.0	584.1 ± 89.1	326.0 ± 102.1	290.1 ± 45.0
CCL5	18780.1 ± 2343.0	21263.9 ± 3589.0	11783.5 ± 3130.2	12851.4 ± 2156.4
CXCL10	1065.2 ± 422.7	319.3 ± 108.7**	68.1 ± 12.4	133.1 ± 25.1**
CX3CL1	440.0 ± 85.6	412.3 ± 72.7	414.2 ± 73.9	507.9 ± 86.5

Values are expressed as mean ± SEM in pg/mL. *p < 0.05, **p < 0.01.

### IVIG Induced Changes in NK Cell Function

**Figure 2:**
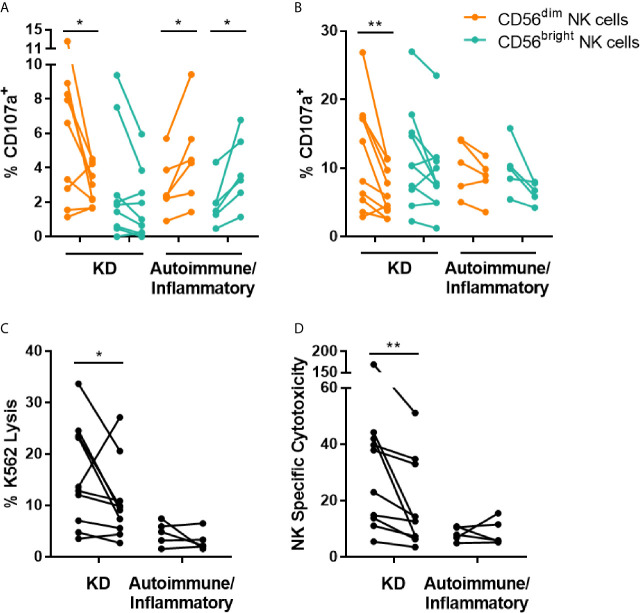
IVIG treatment diminishes NK cell degranulation and NK cell natural cytotoxicity in KD patients. **(A)** PBMCs obtained pre- and post-IVIG infusion from KD patients (n=9-10) and autoimmune/inflammatory patients (n=5-6) were analyzed *ex vivo* by flow cytometry for surface expression of CD107a. **(B–D)** PBMCs were co-cultured with K562 target cells at an E:T ratio of 20:1, followed by immunofluorescent staining and flow cytometric analysis of CD107a **(B)**, K562 lysis **(C)** and NK specific cytotoxicity **(D)**. Graphs show individual paired samples with lines connecting pre-IVIG (left) and post-IVIG (right) data. *p < 0.05; **p < 0.01.

To further examine changes in NK cell function, the effect of IVIG on NK cell degranulation and cytotoxicity was evaluated. *Ex vivo* measurement of the degranulation-associated marker CD107a (21) on the surface of CD56^dim^ NK cells demonstrated a decrease in frequency of CD107a^+^ cells post-IVIG compared to pre-infusion in patients with KD. In contrast, patients with autoimmune/inflammatory diseases were found to have a significantly (p < 0.05) increased percentage of CD107a^+^ cells among both CD56^dim^ and CD56^bright^ NK cells after IVIG (**Figure 2A**). When PBMCs isolated pre- and post-IVIG from KD patients were cultured with K562 target cells, CD107a was expressed on a significantly lower proportion of CD56^dim^ NK cells after IVIG, indicating less killing capacity of these cells (**Figure 2B**). Furthermore, both K562 lysis and NK specific cytotoxicity were significantly decreased in KD patients after IVIG (p = 0.049 and p = 0.002, respectfully; **Figures 2C, D**). CD107a surface expression on CD56^dim^ NK cells was highly correlated with NK cell specific cytotoxicity at the post-IVIG (r = 0.830; p = 0.005) time point in KD patients (**Figure S2**).

### IVIG Decreases NK Cell Pro-Inflammatory Mediator Production

**Figure 3:**
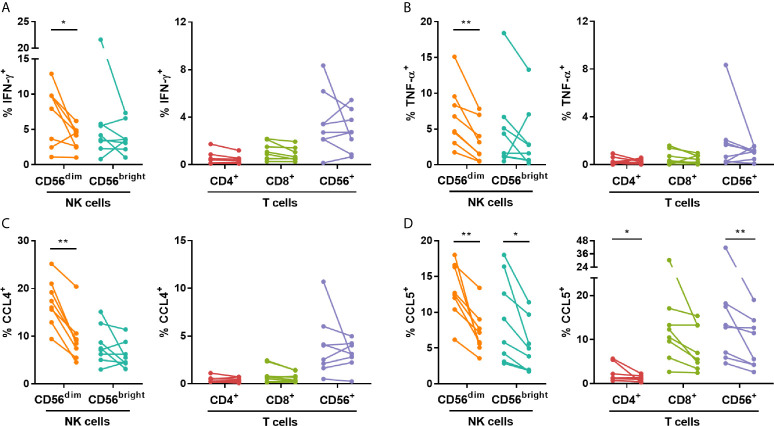
IVIG treatment diminishes NK cell pro-inflammatory mediator production in response to K562 target cells in KD patients. PBMCs obtained pre- and post-IVIG from KD patients (n=8) were rested overnight in 25% autologous plasma obtained at the same time point. PBMCs were then co-cultured with K562 target cells at an E:T ratio of 20:1 in the presence of Brefeldin A, followed by intracellular staining for IFN-γ **(A)**, TNF-α **(B)**, CCL4 **(C)** and CCL5 **(D)**. Graphs show individual paired samples with lines connecting pre-IVIG (left) and post-IVIG (right) data. *p < 0.05; **p < 0.01.

To assess the impact of IVIG on the production of pro-inflammatory cytokine and chemokines known to be produced by NK cells [reviewed in (22)], intracellular cytokine staining was performed after co-culture of PBMCs with K562 target cells. The frequency of CD56^dim^ NK cells producing IFN-γ, TNF-α, CCL4 and CCL5, was significantly lower post-IVIG in patients with KD (**Figures 3A–D**). CD56^bright^ NK cells from KD patients also showed a decrease in the percentage of CCL5^+^ cells (**Figure 3D**). Interestingly, there was substantial CCL5 production by the T cells in KD patients and this was decreased in CD4^+^ and CD56^+^ T cells post-IVIG (**Figure 3D**). CXCL8 and GM-CSF were produced by a smaller proportion of cells and no difference was observed in the production of these mediators except for CXCL8 which was produced by a larger proportion of CD56^+^ T cells in KD patients post-IVIG (**Figure S3**). Due to insufficient sample numbers, autoimmune/inflammatory PBMC mediator production was not assessed.

### IVIG Induced Changes in NK Cell Activating and Inhibitory Receptors

**Figure 4:**
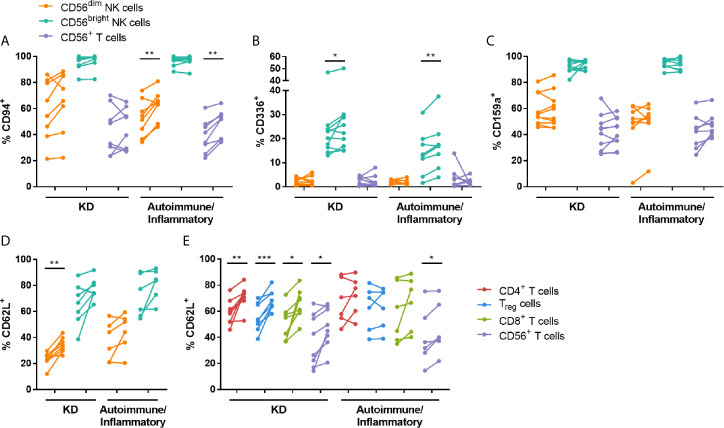
High dose IVIG infusion upregulates the expression of NK receptors and CD62L. PBMCs obtained pre- and post-IVIG from patients with KD (n=8-10) or autoimmune/inflammatory diseases (n=6-8) were analyzed by flow cytometry for surface expression of the NK receptors CD94 **(A)**, CD336/NKp44 **(B)**, CD159a/NKG2A **(C)** and the adhesion molecule CD62L **(D, E)**. Graphs show individual paired samples with lines connecting pre-IVIG (left) and post-IVIG (right) data. *p < 0.05; **p < 0.01; ***p < 0.001.

NK cytotoxicity responses are tightly controlled by the balance of signals transmitted through activating and inhibitory receptors on the cell surface [reviewed in (23)]. Therefore, changes in the expression of selected NK-associated receptors were determined on PBMCs obtained pre- and post-IVIG. The percentage of CD56^dim^ NK cells and CD56^+^ T cells expressing the NKG2 family co-receptor CD94 was increased post-IVIG in autoimmune/inflammatory patients, and the proportion of CD56^bright^ NK cells expressing the activating receptor CD336 was increased post-IVIG in patients with KD and autoimmune/inflammatory diseases (**Figures 4A, B**). No differences were observed in NK cell expression of the activating receptors CD314, CD335 or CD337, or in the inhibitory receptors CD158a, CD158e or CD159a in NK cell or T cell subsets in any patient cohort examined (**Figures 4C** and **S4**).

### IVIG Induced Changes in Trafficking Receptors on NK Cells and T Cells

To broaden our study of IVIG effects on lymphocyte function, surface receptors important for cell trafficking were also analyzed pre- and post-IVIG. CD62L, an adhesion molecule crucial for migration to lymph nodes, was consistently expressed at a significantly higher frequency on CD56^dim^ NK cells and all T cell subsets evaluated post-IVIG in KD patients (**Figure 4D**). In contrast, analysis of PBMCs from autoimmune/inflammatory patients showed that only CD56^+^ T cells expressed CD62L at a higher frequency (**Figure 4D**). No changes in expression of the chemokine receptors CCR2, CCR5, CCR7, CXCR1 or CX3CR1 were found on NK cells (**Figure S5**), however pooled conventional T cells had modest upregulation of CCR2 (1.2-fold increase; p = 0.018) and CCR5 (1.2-fold increase; p = 0.039) post-IVIG in KD patients (data not shown).

### Effect of IL-2 Stimulation on NK and T Cell Subsets Pre- and Post-IVIG

**Figure 5:**
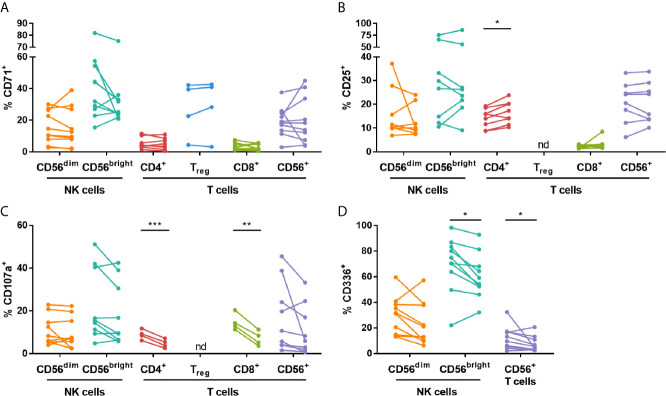
High dose IVIG infusion diminishes CD107a and CD336 expression, and enhances expression of CD25 on various lymphocytes subsets in IL-2-stimulated KD patient PBMCs. PBMCs isolated pre- and post-IVIG from KD patients were stained after 3 days of stimulation with 30 U/mL IL-2 in 50% autologous plasma obtained when PBMCs were collected. PBMCs were analyzed by flow cytometry for surface expression of CD71 **(A)**, CD25 **(B)**, CD107a **(C)** and CD336/NKp44 **(D)**. Graphs show individual paired samples with lines connecting pre-IVIG (left) and post-IVIG (right) data (n=8-10 patients for all markers and subsets except for CD71 on T_reg_ cells and CD107a on CD4^+^ and CD8^+^ T cells where n=4). *p < 0.05; **p < 0.01; ***p < 0.001; nd, not determined.

The effect of IVIG on NK cell and T cell stimulation with low dose (30 U/ml) IL-2 was examined. The most consistent changes occurred in KD patients and are highlighted in **Figure 5**. IVIG therapy did not have a significant effect on the frequency of NK cells expressing CD71 or CD25 following IL-2 stimulation but it did cause upregulation of CD25 on CD4^+^ T cells (**Figure 5B**). In contrast, there was a decrease in CD107a expression on CD4^+^ and CD8^+^ T cells after IVIG treatment (**Figure 5C**). IL-2 stimulation of pre-IVIG PBMCs induced significant, comparable upregulation of the proliferation marker CD71 and the activation marker CD25 on multiple lymphocyte subsets by 3.7-9.5-fold and 3.1-6.3-fold, respectively, compared to unstimulated fresh PBMCs (**Table S2**).

Analysis of NK receptors after IL-2 stimulation demonstrated that the activating receptor CD336 was significantly decreased on CD56^bright^ NK cells and CD56^+^ T cells after IVIG treatment (**Figure 5D**). No significant changes occurred when comparing pre- and post-IVIG PBMCs for expression of CD94, the activating receptors CD314, CD335 and CD337, or the inhibitory receptors CD158a, CD158e and CD159a (data not shown).

Trafficking receptor expression on IL-2 stimulated PBMCs was also determined. No substantial differences were observed in KD patient NK cell and T cell subsets pre- and post-IVIG when assessing CCR2, CCR5, CCR7, CD62L, CXCR1 and CX3CR1 (data not shown). Measurement of selected cytokines secreted during IL-2 stimulation of PBMCs from KD patients showed that after IVIG therapy there was an 87% decrease in IFN-α, a 16% decrease in CX3CL1, and a 98% decrease in GM-CSF production, but IL-33 levels increased by 2.2-fold, all of which were statistically significant (**Table 4**). Several other pro- and anti-inflammatory cytokines assayed were unchanged.

**Table 4:** Cytokine levels in supernatants from KD PBMCs stimulated with IL-2 and *Candida*.

Analyte	IL-2 Stimulation (n = 6)		*Candida* Stimulation (n = 5)	
	Pre-IVIG	Post-IVIG	Pre-IVIG	Post-IVIG
IL-1β	172.5 ± 133.1	57.8 ± 36.4	43.5 ± 17.0	666.6 ± 359.8
IL-4	2.2 ± 0.3	4.3 ± 1.8	1.2 ± 0.2	1.4 ± 0.2
IL-6	5288.4 ± 3156.2	5628.5 ± 5124.6	18904.9 ± 7812.0	42501.9 ± 11518.8
IL-7	7.2 ± 3.3	6.9 ± 2.9	5.2 ± 3.3	5.1 ± 2.9
IL-10	11.4 ± 8.2	6.2 ± 2.6	2.0 ± 1.8	7.6 ± 1.2
IL-12p70	2.7 ± 0.3	2.6 ± 0.6	1.5 ± 0.5	2.6 ± 0.4
IL-13	73.4 ± 17.6	216.2 ± 177.7	3.5 ± 1.3	19.4 ± 16.6
IL-15	6.3 ± 2.0	5.1 ± 1.2	4.2 ± 1.2	4.3 ± 1.1
IL-17F	85.3 ± 70.8	96.4 ± 63.7	20.4 ± 12.7	1113.9 ± 695.5
IL-33	0.5 ± 0.2	1.1 ± 0.3*	1.1 ± 0.2	2.3 ± 0.8
IFN-α	80.7 ± 68.2	10.4 ± 5.9*	15.5 ± 5.5	6.0 ± 1.7
IFN-γ	78.6 ± 19.4	173.2 ± 85.8	2.4 ± 1.2	6.0 ± 2.2
GM-CSF	153.7 ± 69.7	3.5 ± 1.5*	155.7 ± 112.8	50.1 ± 35.7
Granzyme B	6339.8 ± 1804.9	12393.5 ± 3431.6	697.0 ± 240.9	539.7 ± 234.8
PDGF-AB/BB	327.5 ± 87.9	292.9 ± 65.1	343.1 ± 97.1	299.0 ± 98.1
CCL5	7490.4 ± 2479.0	5107.5 ± 828.2	4037.1 ± 2702.0	2632.8 ± 756.9
CXCL10	609.4 ± 143.8	622.7 ± 198.6	388.7 ± 129.9	174.6 ± 41.2
CX3CL1	305.6 ± 28.2	255.5 ± 30.7*	360.7 ± 53.2	269.1 ± 61.1

Values are expressed as mean ± SEM in pg/mL. *p < 0.05.

### Effect of Antigen Stimulation on NK and T Cell Subsets Pre- and Post-IVIG

**Figure 6:**
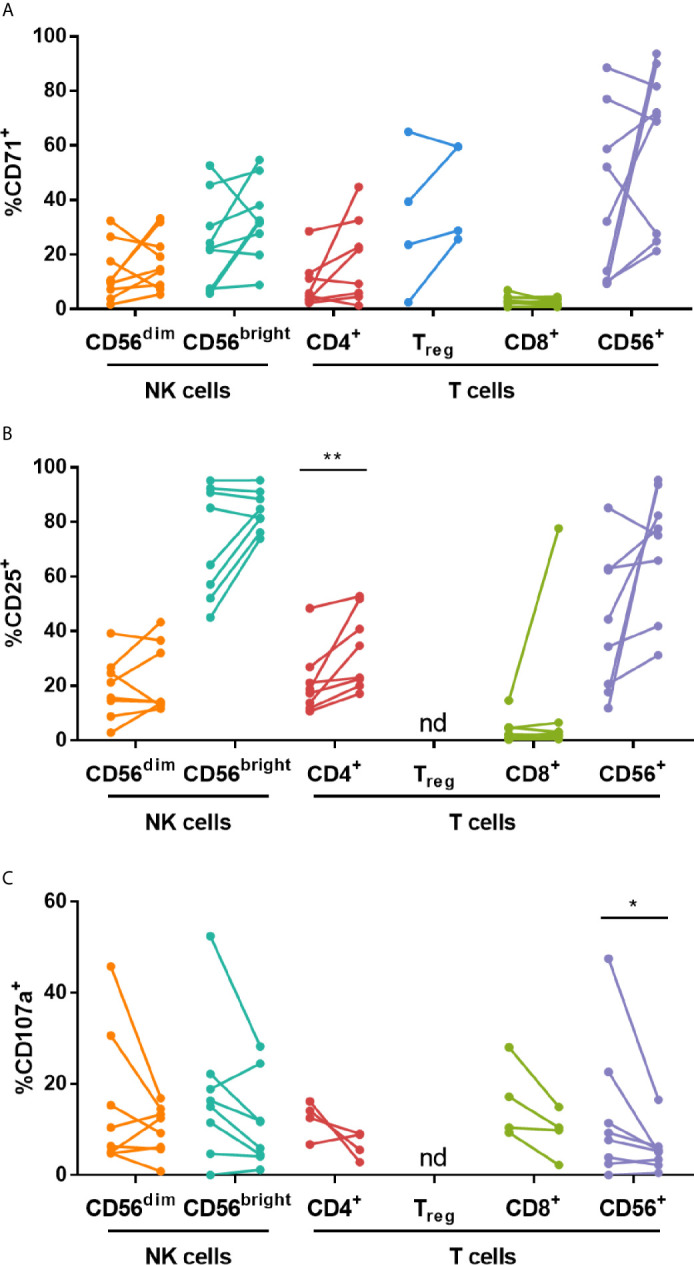
High dose IVIG treatment enhances CD25 expression on CD4^+^ T cells and decreases CD107a expression on CD56^+^ T cells in *Candida* stimulated KD patient PBMCs. PBMCs isolated pre- and post-IVIG from KD patients were stained after 6 days of stimulation with *Candida* antigen in 50% autologous plasma obtained when PBMCs were collected. PBMCs were analyzed by flow cytometry for surface expression of CD71 **(A)**, CD25 **(B)** and CD107a **(C)**. Graphs show individual paired samples with lines connecting pre-IVIG (left) and post-IVIG (post) data (n=8-9 patients for all markers and subsets except for CD71 on T_reg_ cells and CD107a on CD4^+^ and CD8^+^ T cells where n=4). *p < 0.05; **p < 0.01; nd, not determined.

PBMCs were cultured with *Candida* extract for six days as an antigenic stimulus, and the largest changes occurred in KD patients, as discussed below. *Candida* stimulation resulted in upregulation of CD71 and CD25 on multiple lymphocyte subsets by 2.5-16.1-fold and 4.0-7.5-fold, respectively, compared to *ex vivo* levels of expression (**Table S2**). IVIG did not significantly affect the expression of CD71, however the proportion of CD4^+^ T cells expressing CD25 was higher (**Figures 6A, B**). Additionally, CD107a was expressed on a lower percentage of CD56^+^ T cells in post-IVIG PBMCs (**Figure 6C**). NK receptor expression analysis showed that a higher frequency of CD56^bright^ NK cells expressed the inhibitory receptor CD159a post-IVIG (pre-IVIG, 83.7 ± 2.6; post-IVIG, 91.9 ± 1.7; p = 0.033; n=9). Expression of CD94, the activating NK receptors CD314, CD335, CD336, CD337, and NK inhibitory receptors CD158a, CD158e and CD159a and chemokine receptors after *Candida* stimulation was not affected by IVIG treatment (data not shown). Finally, the levels of cytokines in the supernatants of *Candida* antigen-stimulated PBMCs did not show any significant differences between pre- and post-IVIG samples (**Table 4**).

## Discussion

IVIG is an effective treatment for certain diseases presenting as immune dysregulation [reviewed in (1)]. Many studies have examined the mechanisms by which IVIG down-modulates inflammation and autoimmune processes, yet our understanding is still incomplete (5, 6). NK cells are important in innate immunity and in immune regulation. Surface expressed activating Fc receptors enable NK cells to recognize IgG bound to target cells and become activated. Multiple studies have evaluated changes in NK cells induced by IVIG, but no consensus has been reached, and there is a paucity of information on the role of NK cells in the resolution of inflammation in response to IVIG treatment in diseases involving immune dysregulation. The current study investigated the phenotypic and functional changes in CD56^dim^ and CD56^bright^ NK cells, as well as T_reg_ cells and T cells expressing CD4, CD8 and CD56 after IVIG treatment of patients with KD and autoimmune/inflammatory conditions.

We observed that in autoimmune/inflammatory patients, there was a decrease in the frequency of CD56^dim^ NK cells among PBMCs (**Figure 1**), which is in agreement with other studies on women with recurrent miscarriage (10, 24–26), CIDP patients (27) and multiple sclerosis and peripheral neuropathy patients (12). This decrease in CD56^dim^ NK cell frequency did not occur in KD patients, however CD56^dim^ NK cells have been demonstrated to be lower in frequency compared to normal donors in KD patients at the onset of acute disease (28, 29). We also saw an increase in the percentage of CD71^+^ CD56^dim^ NK cells after IVIG indicating increased proliferation of CD56^dim^ NK cells in this group of patients (**Figure 1**). Previously, our group showed that stimulation of normal donor PBMCs *in vitro* with submitogenic concentrations of IL-2, IL-15 or IL-18, in the presence of IVIG induced selective NK cell proliferation (30). IVIG was also shown to drive NK cell proliferation *in vivo* in a mouse model of graft-versus-host disease, in which it was associated with suppression of the disease (31). However, ours is the first study to show IVIG therapy causing NK proliferation in the context of immune dysregulation.

After IVIG treatment of KD patients, there were divergent changes in NK cell subsets. The proportion of CD56^bright^ NK cells expressing the activating receptor CD336 was higher (**Figure 4**) but CD56^dim^ NK cells exhibited lower responses to K562 target cells resulting in decreased cytotoxicity (**Figure 2**). This is the first publication to demonstrate an IVIG-induced change in CD336 expression in any clinical setting. CD336 was also expressed on a higher frequency of CD56^bright^ NK cells in autoimmune/inflammatory patients, and therefore this was a more global IVIG effect. However, NK cell cytotoxicity to K562 cells was not altered by IVIG in these patients, whose PBMCs had relatively low cytotoxic activity prior to IVIG.

The percentage of CD56^dim^ NK cells expressing CD94 was also increased after IVIG, and this occurred specifically in autoimmune/inflammatory patients. This has been demonstrated also following IVIG treatment in women with recurrent spontaneous abortions (32), however, our study is the first to show such an effect in immune dysregulation and hyperinflammation where there is active inflammation. NK cell function is governed by a combination of signals transmitted through both activating and inhibitory receptors [reviewed in (23)]. CD94 is a co-receptor for the NKG2 C-type lectin receptors, which include both activating and inhibitory receptors, and increased expression of CD94 could increase recognition of CD94 heterodimer ligands. Ahmadi et al. demonstrated that a low dose of IVIG upregulated inhibitory receptors and decreased activating receptors, which coincided with a decrease in natural cytotoxicity by PBMCs from women with recurrent spontaneous abortion (10). Additionally, Mausberg et al. found that multiple NK receptors, including CD94, were downregulated at the transcriptional level in PBMCs post IVIG infusion of patients with chronic inflammatory demyelinating polyneuropathy (CIDP) (33). After a high dose of IVIG, we did not observe similar changes in the receptors but did find a marked decrease in both the expression of the granule exocytosis marker, CD107a, and in cytotoxicity that was most striking in the clinically homogenous KD group. In addition, we also demonstrate for the first time a significant decrease in pro-inflammatory mediator production including IFN-γ, TNF-α, CCL4 and CCL5 by these CD56^dim^ NK cells (**Figure 3**). Possible sources of discordances between results in these studies may be differences in patient age, medical condition and treatments received prior to the studies.

The CD56^bright^ NK cells were also affected by IVIG treatment. In KD patients a higher proportion of CD56^bright^ NK cells were CD25^+^ compared to CD56^dim^ NK cells (**Figure 1**), and the frequency of CD56^bright^ NK cells expressing CD25 was increased further by IVIG. This suggests an enhancement of CD56^bright^ NK cell function. However, there was no difference in CD71, CD107a and cytokine secretion by these cells after IVIG in KD patients. In contrast, analysis of CD56^bright^ NK cells in autoimmune/inflammatory patients showed that a higher proportion of these cells were CD71^+^ (**Figure 1C**) and CD107a^+^ (**Figure 2A**). When these CD56^bright^ NK cells were stimulated *in vitro* with IL-2 or were cultured in the presence of *Candida*, they responded by proliferating (upregulated CD71) and increasing CD25 expression (**Table S2**). However, none of these changes were significantly affected by IVIG. These results indicate that IVIG diversely affects CD56^bright^ NK cells depending on the disease state.

The effects of IVIG treatment on various T cell subsets was also examined. Our data showed that T_reg_ cells were increased in frequency of PBMCs in patients with KD after IVIG infusion. Multiple studies have also demonstrated that IVIG promotes T_reg_ cell expansion and activation in KD (34, 35), in women with recurrent miscarriages (36), and in a murine model of multiple sclerosis, i.e. experimental autoimmune encephalomyelitis (EAE) (37). T_reg_ cells are crucial for the maintenance of immune homeostasis, and decreased T_reg_ frequency has been demonstrated in KD patients (35, 38, 39). In the EAE mouse model, IVIG-induced resolution of inflammation and disease activity was dependent on T_reg_ cell activation and expansion, but NK cells were required for this response to IVIG (37). We hypothesize that in the context of hyperinflammation, when NK activating cytokines such as IL-2, IL-15 and IL-18 are being over produced, IVIG may augment NK cell proliferation to these cytokines, as previously shown by us *in vitro* (24). These activated NK cells may promote T_reg_ cell expansion and lead to a down regulation of inflammation.

Our studies also assessed the effects of IVIG on CD4^+^, CD8^+^ and CD56^+^ T cells mainly as a comparative population for the NK cell changes due to IVIG. Similar to the CD56^dim^ NK cells, there was a marked decrease in the production of CCL5 by CD4^+^ and CD56^+^ T cells in KD patients and an increase in the number of CXCL8^+^ CD56^+^ T cells, but no change occurred in the other four mediators examined (**Figures 3** and **S3**). Interestingly, IVIG elevated the proportion of CD4^+^ T cells expressing CD25, but decreased the frequency of CD107a^+^ cells among both CD4^+^ and CD8^+^ T cells when PBMCs were stimulated with IL-2 (**Figure 5**). These results suggest that *in vivo*, in the presence of IL-2, IVIG may down-modulate granule exocytosis as well as secretion of certain inflammatory cytokines by multiple T cell subsets. The secretion of cytokines by PBMCs during culture with IL-2 in post-IVIG plasma, as measured by levels of IFN-α, CX3CL1 and GM-CSF in supernatants, was also decreased, and strikingly so in the case of GM-CSF (**Table 4**). To our knowledge no other studies have examined responses to such stimuli with PBMCs cultured in a concentration of autologous plasma (50%) similar to that which lymphocytes are exposed to before and after IVIG treatment of patients with KD and autoimmune/inflammatory diseases. In this way the infused IgG and any *in vivo* generated mediators present in plasma would be in the culture, available to exert their effects on the cellular responses *ex vivo*.

Across multiple studies with various conditions, the general consensus is that IVIG inhibits NK cell cytotoxicity. This was shown *in vitro* using IVIG-treated PBMCs from normal donors (12, 13, 40, 41), and in PBMCs after IVIG infusion of women with recurrent miscarriages (10, 14, 15, 25, 42), patients with immune thrombocytopenia (11) and CIDP (27). Our findings in KD patients agree with these reports (**Figure 2**). A few studies have shown either no change (43) or augmentation (14, 31) of NK cell cytotoxicity after IVIG including one focusing on KD patients by Finberg et al. (16), which is discordant with our findings. The reason for this is not clear but in their study the patient’s blood NK cell numbers increased markedly after IVIG, in contrast to our findings. If the actual NK cell to K562 target ratio in the assay is not taken into account, as it was in our study, an apparent increase in cytotoxicity might be seen. Investigations of blood transcription profiles and pathway analysis demonstrated that the expression of NK cell cytotoxicity genes is altered in acute KD patients compared to healthy controls (44, 45). Our data show that NK cell functions that are likely affected by systemic inflammation in KD are modulated by IVIG.

NK cells are also potent sources of both pro-inflammatory and anti-inflammatory cytokines [reviewed in (22)]. Our results demonstrated that IVIG treatment down-modulated target cell-induced production of multiple pro-inflammatory cytokines, including IFN-γ, TNF-α, CCL4 and CCL5, by CD56^dim^ NK cells in KD patients (**Figure 3**). Ebbo et al. also demonstrated that K562-induced IFN-γ production is reduced by IVIG in immune thrombocytopenia patients (11), and our study in KD patients expands this concept by showing a broader inhibition of cytokine responses. The decrease in the secretion of these mediators suggests that this may be an important component of the effect of IVIG on NK cells quite independent of their decreased cytotoxicity. This finding deserves further investigation to understand the effects of IVIG on NK cells.

An effect of IVIG that was common to multiple NK cell and T cell subsets was an increase in the frequency of cells expressing CD62L *in vivo*, which largely occurred in KD patients (**Figure 4**). This has been previously demonstrated in human myeloid DCs (46) and murine CD8^+^ T cells (47), and does not appear to occur when IVIG is administered at low doses (24). CD62L is an important adhesion molecule involved in trafficking from the blood into lymphoid tissues [reviewed in (48)]. Upon activation, leukocytes can shed CD62L from the surface, resulting in increased soluble CD62L. In KD, soluble CD62L in plasma does not increase after IVIG infusion (49, 50), indicating that the activation status of leukocytes is not substantially increased by IVIG, and this aligns with our observation that cell surface CD62L is elevated after IVIG. Furthermore, measurements of cytokines in patient plasma revealed a decrease in the levels of IL-6 and CXCL10 in the KD cohort (**Table 3**), of which the latter has also been demonstrated by Ko et al. and has been proposed as a predictor of KD (51). These pro-inflammatory mediators may contribute to lymphocyte activation during systemic inflammation, and these declining plasma levels coincided with increased lymphocyte CD62L expression post-IVIG. It is not known whether the upregulated surface CD62L alters lymphocyte trafficking, which is highly dependent on chemokines as well, but CD62L is one prerequisite for lymphocyte trafficking to lymphoid tissues. We did not observe substantial changes in expression of various chemokine receptors, with the exception of a minor increase in the proportion of T cells expressing CCR2 and CCR5. These results suggest that IVIG may impact lymphocyte trafficking, but further investigation of this is required.

Immune dysregulation presenting as auto- or hyper-inflammation occurs in a variety of conditions, and IVIG is an effective treatment in some of these disorders. A common thread among these debilitating diseases is an overactive immune system resulting in cellular and tissue injury due to inflammation (e.g., aneurysms in KD; demyelination in CIDP). The processes that lead to tissue damage involve a complex network of leukocytes, tissue-resident cells and pro-inflammatory mediators. The diseases treated by IVIG have different etiologies and likely diverse immunologic mechanisms, and IVIG can exert beneficial effects in all the conditions studied here. Most of our observations were specific to one cohort, but in both KD and autoimmune/inflammatory patients the activating NK receptor CD336 was expressed on a higher frequency of CD56^bright^ NK cells (**Figure 4B**). In other measurements, KD patients contrasted with autoimmune/inflammatory patients, such as the expression of CD107a on CD56^dim^ NK cells *in vivo* (**Figure 2A**) and the change in plasma CXCL10 (**Table 3**). These apparent differences may be due to the distinct immunopathological processes that are present in each of the cohorts. Representative of this, KD patients had higher levels of plasma IL-6 and CXCL10 at baseline compared to autoimmune/inflammatory patients. It should be noted that there was a clear difference in age and gender between the two cohorts (**Tables 1** and **2**), so it is possible that the findings described here may be affected by those factors.

Overall, these results demonstrate that IVIG induced multiple phenotypic and functional changes in NK cell and T cell subsets, mainly promoting resolution of inflammation, in patients treated for immune dysregulation. Some of these changes occurred only in KD patients, indicating that IVIG induces disease-specific effects. Our results provide novel insight into the mechanisms of action of IVIG and may lead to new rationale for novel therapeutic applications of IVIG and subcutaneous Ig in immune dysregulation such as autoimmunity and chronic inflammatory conditions.

## Data Availability Statement

The raw data supporting the conclusions of this article will be made available by the authors, without undue reservation.

## Ethics Statement

The studies involving human participants were reviewed and approved by IWK Research Ethics Board, IWK Health Centre. Written informed consent to participate in this study was provided by the participants’ legal guardian/next of kin.

## Author Contributions

SM, SR, AI, TI, and BD contributed to conception and design of the study. SM, SR, JH, and BD carried out the experiments and/or acquired the data. SM, SR, JH, AI, TI, and BD analyzed and interpreted the data. SM and SR performed the statistical analysis. SM wrote the first draft of the manuscript. SM, SR, AI, and TI wrote sections of the manuscript. All authors contributed to the article and approved the submitted version.

## Funding

This study was funded by an investigator-initiated industry grant from CSL Behring.

## Conflict of Interest

Author FK was employed by the company CSL Behring. The authors declare that this study received funding from CSL Behring in the form of an investigator-initiated grant. The funder was not involved in the study design, collection, analysis, interpretation of data or the writing of this article. Author FK read and approved the manuscript.

## Abbreviations

ADCC, antibody-dependent cell-mediated cytotoxicity; CIDP, chronic inflammatory demyelinating polyneuropathy; IFN, interferon; HSA, human serum albumin; IL, interleukin; IVIG, intravenous immunoglobulin; KD, Kawasaki disease; NK, natural killer cell; T_reg_, regulatory T cell.
