# Supplementary material for: High Dose Intravenous IgG Therapy Modulates Multiple NK Cell and T Cell Functions in Patients With Immune Dysregulation
Source: Front Immunol. 2021 May 19;12:660506. doi: 10.3389/fimmu.2021.660506 (PMC8170153; doi:10.3389/fimmu.2021.660506)
Supplement: Supplementary file 1 [file DataSheet_1.pdf]

Table S1. Antibodies and reagents used for immunofluorescent staining

Marker	Fluorochrome	Clone	Supplier
CD3	APC-Fire750	UCHT1	BL
CD3	BV510, BV650	OKT3	BL
CD4	PerCP-Cy5.5, BV605, FITC	OKT4	BL
CD8	BV510, BV785	SK1	BL
CD8	FITC	OKT8	To
CD8	APC	RPA-T8	BD
CD11b	BV785, PE-Cy7	ICRF44	BL
CD14	FITC	63D3	BL
CD16	BV605	3G8	BD
CD25	BV421	2A3	BD
CD56	BB515	B159	BD
CD56	APC-R700	NCAM16.2	BD
CD62L	BV605	DREG-56	BL
CD71	BV711	M-A712	BD
CD94	FITC, PerCP-Cy5.5	DX22	BL
CD107a	BV510	H4A3	BD
CD107a	Alexa647, PE-Cy7	H4A3	BL
CD127	APC, BV650	A019D5	BL
CD127	APC	A019D5	To
CD158a	PE-Vio770	REA284	MB
CD158e1	APC	DX9	MB
CD159a	PE	REA110	MB
CD314	PE-CF594	1D11	BD
CD335	BV786	9E2	BD
CD335	BV421	9E2	BL
CD336	BB515	P44-8	BD
CD337	BV605	P30-15	BD
CCR2	APC, PerCP-Cy5.5	K036C2	BL

Marker	Fluorochrome	Clone	Supplier
CCR5	PE-Dazzle594	J418F1	BL
CCR7	APC, BV650	G043H7	BL
CXCR1	PE-Cy5	8F1	BL
CX3CR1	BV421	2A9-1	BD
FoxP3	PE	206D	BL
GM-CSF	PE	BVD2-21C11	BL
IFN-γ	BV711, PE	4S.B3	BL
IL-8	PE-CF594	G265-8	BD
MIP-1β	PerCp-Cy5.5	D21-1351	BD
MIP-1β	APC	REA511	MB
RANTES	BV421	2D5	BD
TNF	PE-Cy7	MAb11	BL
TNF	APC-Vio770	cA2	MB
Viability Dye	eFluor506, APC-eFluor780	-	eBio

BD, BD Biosciences; BL, BioLegend; eBio, eBioscience; MB, Miltenyi Biotec; To, Tonbo Biosciences.

Table S2. Expression of CD71 and CD25 *ex vivo* and after stimulation of KD patient PBMCs isolated pre-IVIG

			CD71 (%)		CD25 (%)	
Stimulus	Population	Subset	*ex vivo*	Post-stimulation	*ex vivo*	Post-stimulation
IL-2	NK cells	$CD56^{dim}$	3.7 ± 1.0	15.6 ± 3.1**	2.6 ± 0.5	16.2 ± 3.5**
(3 days)		$CD56^{bright}$	4.3 ± 0.7	40.8 ± 6.0***	18.4 ± 4.8	33.7 ± 8.2
	T cells	$CD4^{+}$	3.6 ± 1.2	5.4 ± 1.2	16.9 ± 2.2	14.1 ± 1.4*
		T_{reg}	20.2 ± 5.5	27.1 ± 7.6	nd	nd
		$CD8^{+}$	2.6 ± 0.7	3.5 ± 0.7	2.9 ± 1.0	2.1 ± 0.2
		$CD56^{+}$	5.2 ± 1.8	19.2 ± 2.8***	6.7 ± 1.5	20.8 ± 2.9**
Candida	NK cells	$CD56^{dim}$	2.5 ± 0.7	13.7 ± 3.6**	2.6 ± 0.5	19.2 ± 3.8**
(6 days)		$CD56^{bright}$	3.9 ± 0.8	24.1 ± 5.9**	18.4 ± 4.8	72.7 ± 6.7****
	T cells	$CD4^{+}$	3.6 ± 1.2	9.1 ± 2.9*	16.9 ± 2.2	21.1 ± 4.0
		T_{reg}	20.2 ± 5.5	32.6 ± 11.4	nd	nd
		$CD8^{+}$	2.6 ± 0.7	3.3 ± 0.6	2.9 ± 1.0	3.8 ± 1.6
		$CD56^{+}$	2.7 ± 0.9	42.8 ± 10.2**	6.7 ± 1.5	42.4 ± 8.6**

Data are expressed as mean ± SEM of 8-10 patients except T_{reg} cells (n=4). *p < 0.05, **p < 0.01, ***p < 0.001, ****p < 0.0001, compared to *ex vivo,* fresh PBMCs. nd, not determined.

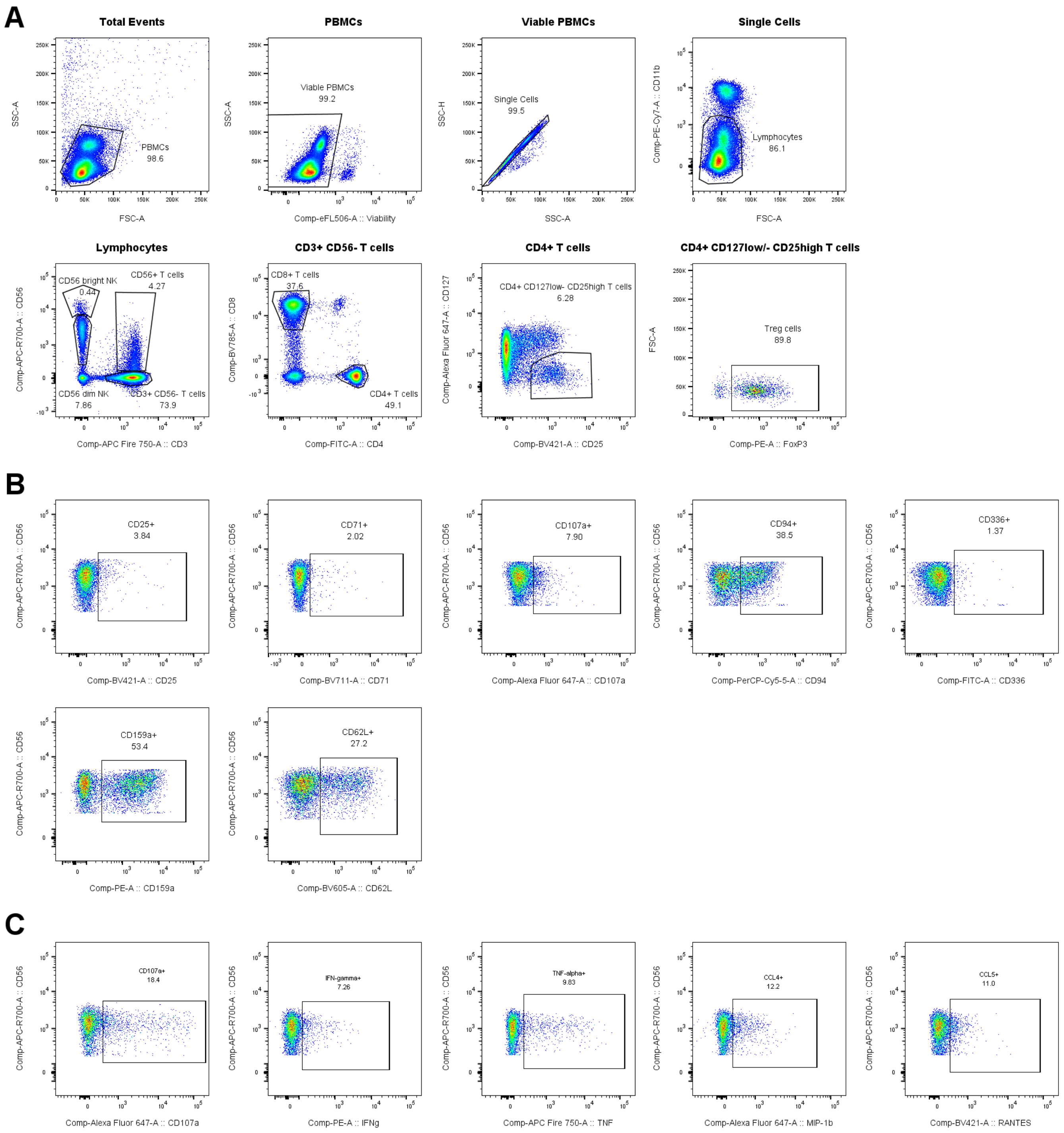

Figure S1. Representative gating strategy for flow cytometric analysis of NK cell and T cell subsets. PBMCs were identified based on size and granularity. After performing live cell determination using viability dye and doublet discrimination, lymphocytes were identified by excluding CD11b^{high} cells. CD3 and CD56 co-staining enabled identification of CD56^{dim} and CD56^{bright} NK cell subsets, CD56^{+} T cells and CD3^{+}CD56^{-} T cells. T cells were further divided into CD4^{+} and CD8^{+} T cell subsets, and T$_{reg}$ cells were identified based on the CD4^{+}CD127$^{low/-}$CD25^{high}FoxP3^{+} phenotype. A representative example of the gating performed on CD56^{dim} NK cells that were freshly thawed (B) or co-cultured with K562 target cells (C) is shown for selected markers.

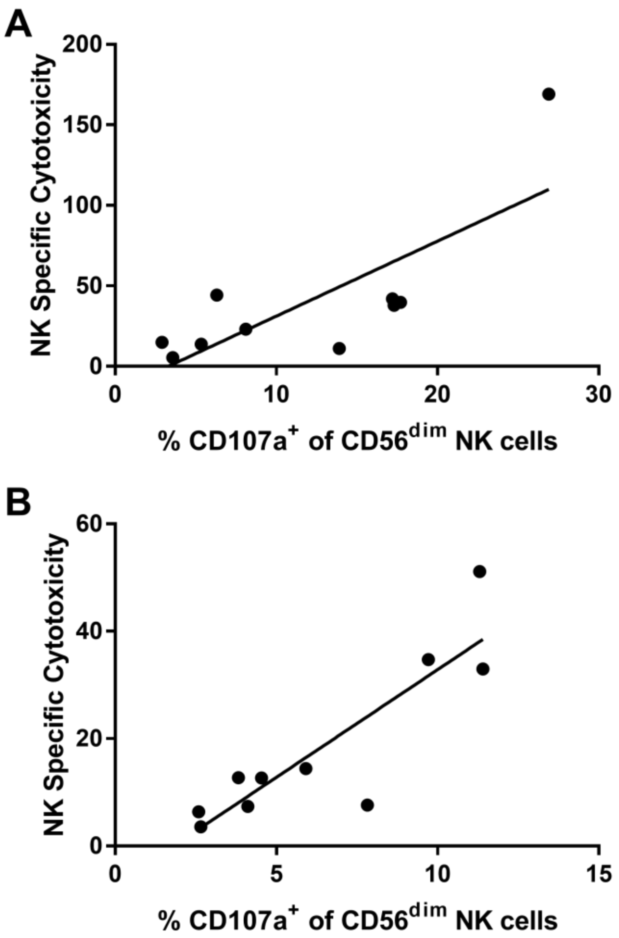

Figure S2. CD107a is highly correlated post-IVIG with NK specific cytotoxicity in response to K562 cells. PBMCs obtained pre-IVIG (A) and post-IVIG (B) from KD patients (n=10) were co-cultured with K562 target cells at an E:T ratio of 20:1, followed by immunofluorescent staining and flow cytometric analysis. Spearman's correlation and linear regression analyses were performed to evaluate the correlation between CD107a expression on $CD56^{dim}$ NK cells and NK specific cytotoxicity at the pre-IVIG ($r = 0.636$, $p = 0.054$) and post-IVIG ($r = 0.830$, $p = 0.005$) time points.

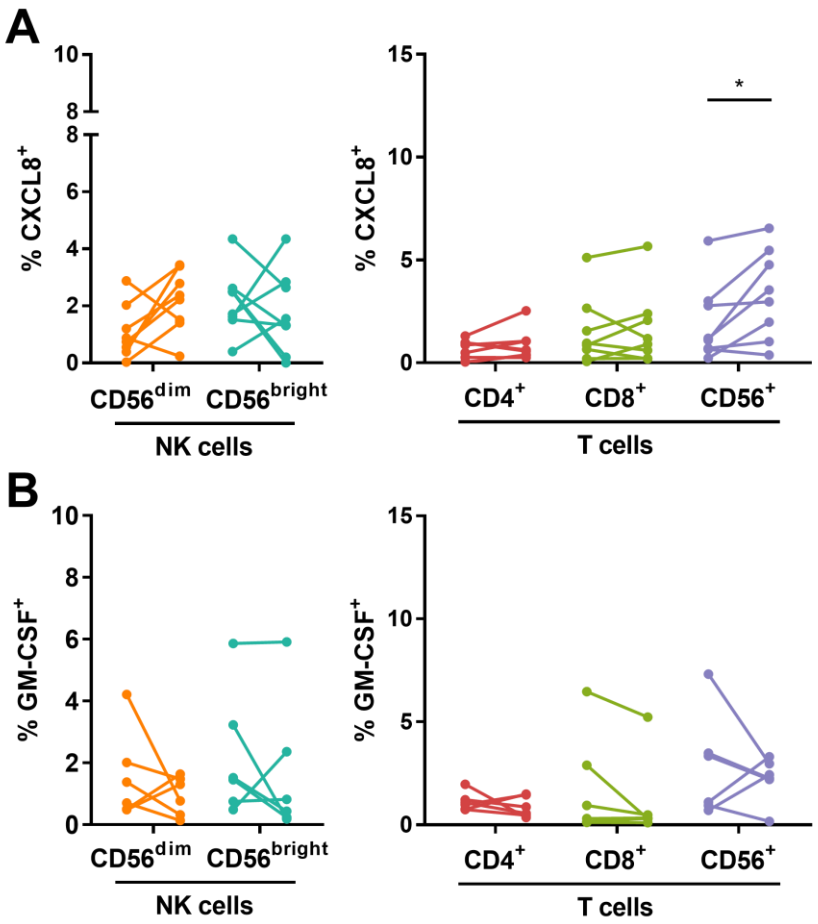

Figure S3. IVIG did not induce substantial changes in CXCL8 and GM-CSF production in response to K562 cells. PBMCs obtained pre- and post-IVIG from KD patients (n=6-8) were rested overnight in 25% autologous plasma obtained at the same time point. PBMCs were then co-cultured with K562 target cells at an E:T ratio of 20:1, followed by intracellular staining for CXCL8 (A) and GM-CSF (B). Graphs show individual paired samples with lines connecting pre-IVIG (left) and post-IVIG (right) data. *$p < 0.05$.

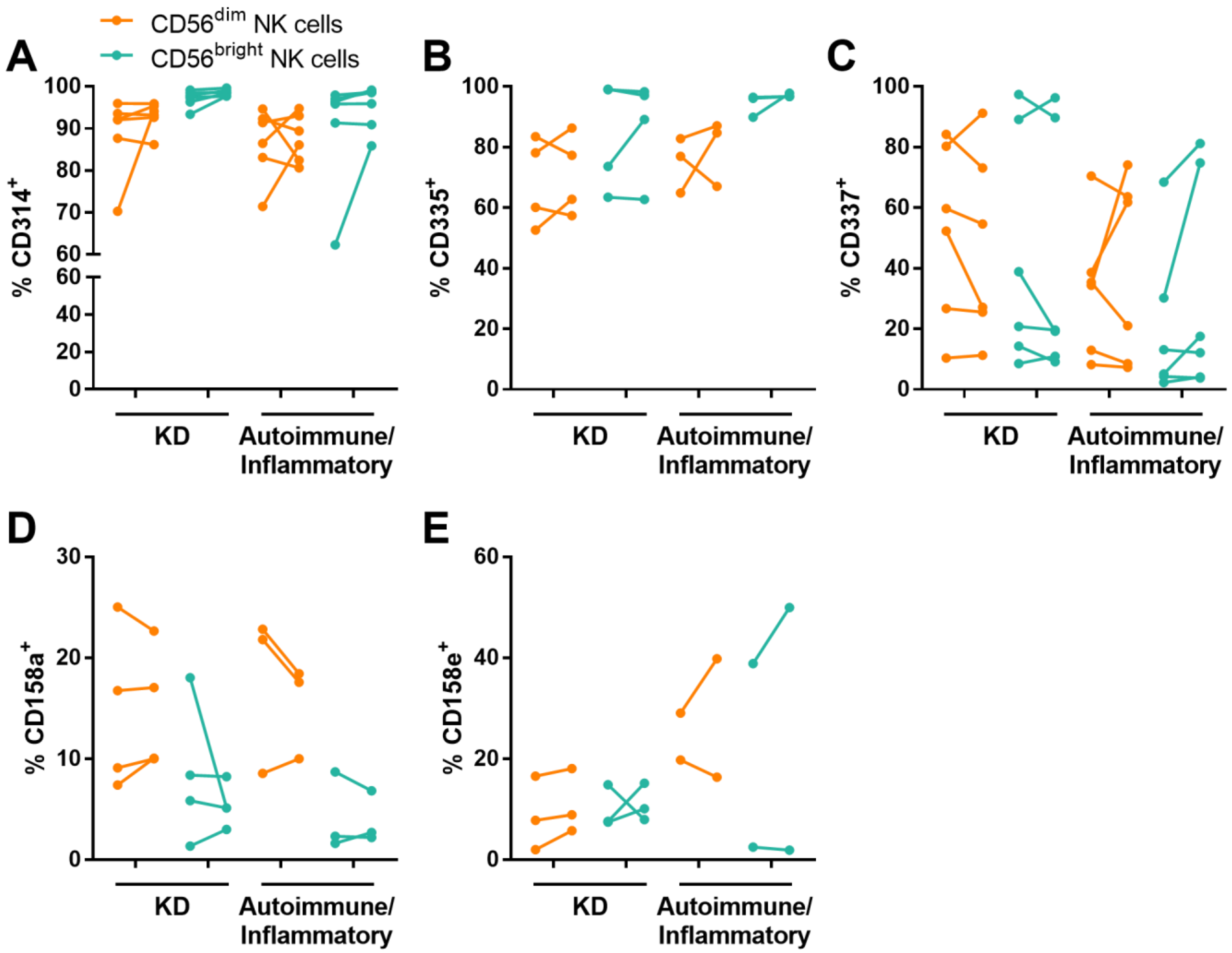

Figure S4. Expression of selected NK receptors was not changed following IVIG infusion. PBMCs obtained pre- and post-IVIG from patients with KD or autoimmune/inflammatory diseases were analyzed by immunofluorescence and multiparameter flow cytometry for surface expression of the activating receptors CD314/NKG2D (A; n=6), CD335/NKp46 (B; n=3) and CD337/NKp30 (C; n=6), and the inhibitory receptors CD158a/KIR2DL1 (D; n=3) and CD158e/KIR3DL1 (E; n=2-3). Graphs show individual paired samples with lines connecting pre-IVIG (left) and post-IVIG (right) data.

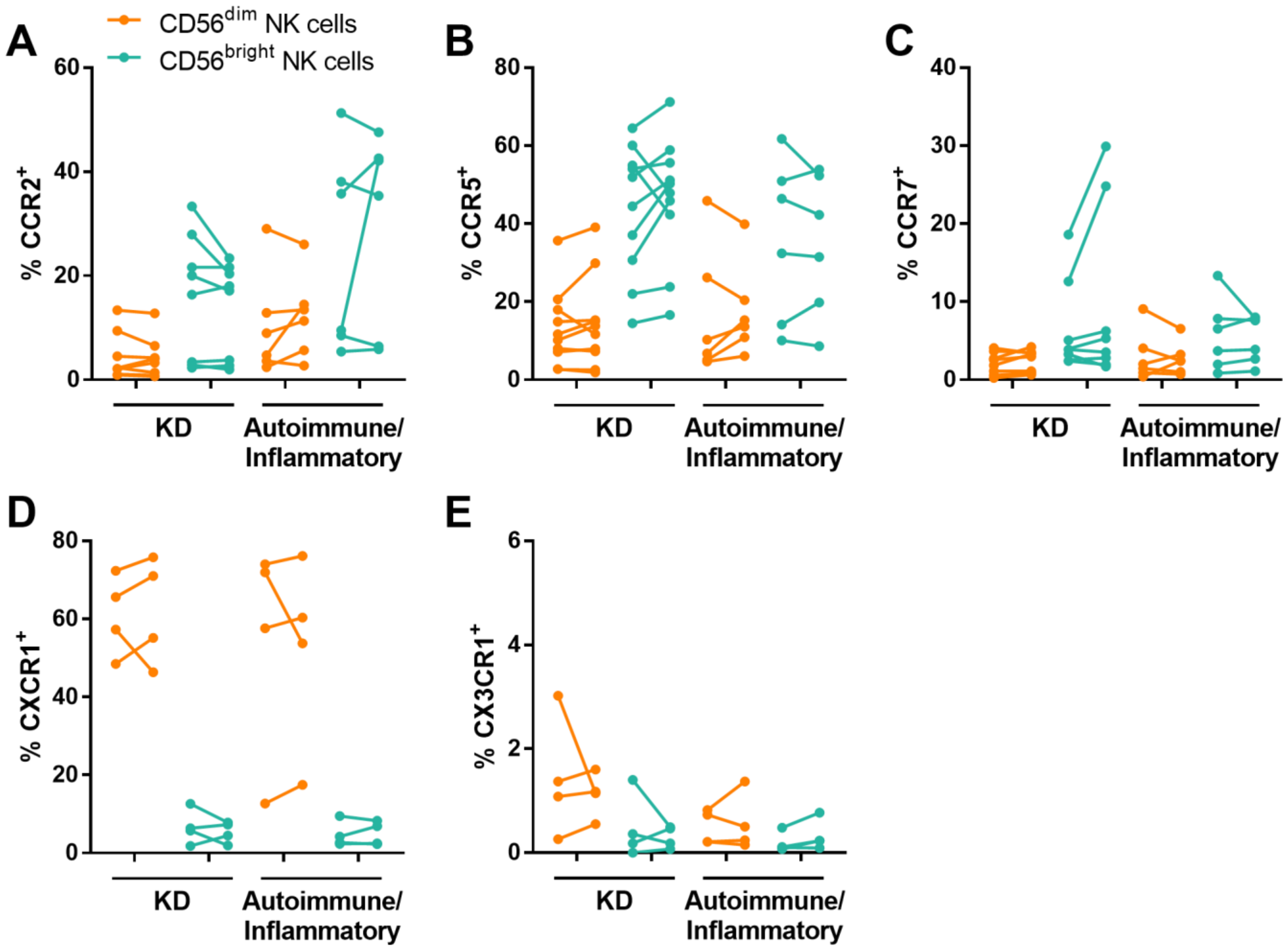

Figure S5. IVIG has little effect on expression of selected chemokine receptors on NK cell subsets. PBMCs obtained pre- and post-IVIG from patients with KD or autoimmune/inflammatory diseases were analyzed by immunofluorescence and multiparameter flow cytometry for surface expression of the chemokine receptors CCR2 (A; n=6-8), CCR5 (B; n=6-10), CCR7 (C; n=6-8), CXCR1 (D; n=4) and CX3CR1 (E; n=4). Graphs show individual paired samples with lines connecting pre-IVIG (left) and post-IVIG (right) data.
